# Risks and Safeguards in Social‐Behavioural Research With Adults With Developmental Disabilities: A Qualitative Systematic Review

**DOI:** 10.1111/jar.70031

**Published:** 2025-03-07

**Authors:** Katherine McDonald, Madison Brodeur, Ariel Schwartz

**Affiliations:** ^1^ Public Health Syracuse New York USA; ^2^ Occupational Therapy MGH Institute of Health Professions Boston Massachusetts USA; ^3^ Institute on Disability University of New Hampshire Durham New Hampshire USA

**Keywords:** developmental disability, inclusion, research ethics, responsible, risks, safeguards

## Abstract

**Background:**

We identified participant‐level risks and safeguards in social‐behavioural research with adults with developmental disabilities.

**Method:**

We conducted a qualitative systematic review on risks and safeguards in peer‐reviewed research with adults with developmental disabilities. We identified research reporting on risks and safeguards published between 2009 and 2023 by searching eight databases and conducting hand searches of reference lists. We conducted thematic analysis using independent data extraction and coding, and investigator triangulation.

**Results:**

From 23 manuscripts, we identified: (1) risks associated with research participation of adults with developmental disabilities (e.g., physical, relational, psychological, and social risks as well as a loss of privacy and confidentiality) and (2) safeguards (e.g., using guiding frameworks, reducing participant burden, securing privacy and confidentiality, and fostering psychological and relational well‐being).

**Conclusions:**

We encourage researchers to foster positive experiences so research participants feel valued and respected, and enjoy having the experience and opportunity to contribute to scientific discoveries.

1

**Summary**

- It is important to take care of people when they are in research by respecting their rights and not hurting them.
- We looked at research studies with adults with developmental disabilities to learn how they might be hurt when they do research and how researchers can keep them safe.
- We learned that many of the ways people might be hurt and can be kept safe may be the same as for people without developmental disabilities. We also learned some things that are specific to doing research with adults with developmental disabilities. These include the importance of taking breaks, protecting people’s privacy and confidentiality, and showing respect.
- We encourage people who do research to create positive experiences. This means research participants feel valued and respected, and enjoy the experience and opportunity to contribute to research.

## 2 Background

Scientific discoveries can advance social and health equity: they allow us to identify and characterise a population, document disparities, and detect policies and practices that can foster opportunities and desired outcomes. Achieving these outcomes requires the inclusion of all people in research (Spong and Bianchi 2018). Yet adults with developmental disabilities are frequently excluded from such opportunities (DeCormier Plosky et al. 2022; Feldman et al. 2014; McDonald et al. 2022), and are simultaneously at risk for inclusion in ways that restrict their rights and threaten their well‐being (McDonald and Kidney 2012; Northway 2014).

Responsible inclusion in research includes the identification of participant‐level research‐related risks and safeguards to mitigate risks and secure individuals' rights and well‐being (Spong and Bianchi 2018). We focus here on participant‐level risks, or the possible negative outcomes research participants may experience from participating (Spong and Bianchi 2018). Risks are contextual, can be psychological, social, legal, economic, and physical in nature, may emerge from the invasion of privacy, loss of confidentiality, and study procedures. Risks also vary in their probability and magnitude of harm. Critically, the identification of risks—based on reliable, substantial evidence and not opinion (National Bioethics Advisory Commission 2002)—can be challenging in social‐behavioural research because they are less identifiable and more subjective than in biomedical research. There is little research about social‐behavioural research risks—both anticipated and experienced (Iltis et al. 2013). Since minimising harm to research participants is paramount, including by leveraging safeguards—strategies researchers can implement to prevent risks and mitigate their negative impacts (The World Medical Association 2013; US Department of Health and Human Services 2018; National Commission for the Protection of Human Subjects of Biomedical and Behavioral Research 1979)—that respond dynamically to potential risks, risk identification is a key element of research protocol development.

Given historic abuses and concerns about vulnerability, research with people with developmental disabilities is highly scrutinised. Notably, ableism can further complicate risk identification. For example, risks to participants with developmental disabilities are often overestimated by researchers and ethics review boards (Snipstad 2022). Further, many strategies to mitigate risks (e.g., requirements for capacity assessments, exclusion from participation) underestimate capacities and restrict rights (Northway 2014; McDonald et al. 2009, 2017, 2024). However, changing social norms increasingly assert the capacities of people with developmental disabilities to make their own decisions and to demonstrate similar resiliency as those without developmental disabilities (McDonald et al. 2017; Buchanan and Warwick 2021; Freedman 2001; Labott and Johnson 2004; McDonald and Keys 2008).

Approaches to risk identification and the implementation of safeguards inform how a population and individuals therein are treated in science. Both contribute to the creation or denial of opportunities for inclusion and respectful, safe treatment. These are important outcomes because responsible inclusion can increase the availability of research evidence for people with developmental disabilities and promote trust, which can promote research participation. To enhance the quality of evidence available to inform risk assessment and selection of safeguards, we conducted a qualitative systematic review to identify participant‐level risks—including those anticipated and those observed—and safeguards in social‐behavioural research with adults with developmental disabilities, with a focus on research procedures (e.g., data collection) and dissemination.

## 3 Methods

### 3.1 Literature Search and Selection

As part of a larger project (Schwartz et al. 2025), we conducted a qualitative systematic review to identify ethical, legal, and social issues (ELSI) in social‐behavioural research with adults with developmental disabilities and for community research partners with developmental disabilities. We searched eight health and social‐behavioural research databases (Web of Science, SCOPUS, ProQuest, PubMed, PsycINFO, ERIC, CINAHL, and ASSIA), working with information specialists to create database‐specific search strings (see Supplemental Table 1). We restricted search criteria to articles published in English in peer‐reviewed journals between January 1, 2009, and June 2, 2022, updating the search in March 2023. This timeframe captures a time of advancing disability rights and increased inclusion of adults with developmental disabilities in research.

**FIGURE 1:**
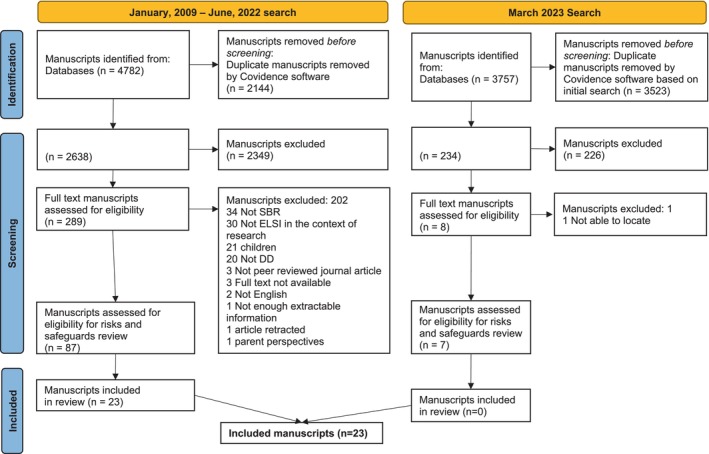
PRISMA diagram.

We input all database search results into Covidence (Covidence n.d.)—a software that organises review activities and automates the removal of duplicate manuscripts. After the automated removal of duplicates, two reviewers (a trained research assistant and one of the two principal investigators, or PIs) independently screened 2638 titles and abstracts to identify eligible manuscripts using the following criteria: (1) peer‐reviewed journals (any article type); (2) content specific to adults with developmental disabilities; and (3) a: addressed ELSI in research participation or b: focused on ethical issues relevant to community research partners. We resolved discrepancies via discussion, with the PIs making final decisions. If it appeared a manuscript might be eligible, we reviewed the full text. The PIs then reviewed full texts (*N* = 289 for the initial search, *N* = 8 for the second search), applying the same criteria and approach. We identified 87 manuscripts for inclusion in the larger study and no additional manuscripts from the second search; our review of reference lists also did not yield any additional manuscripts. For this study, we applied additional eligibility criteria: (1) provided views or data on participant‐level risks and safeguards from the perspectives of researchers, people with developmental disabilities, service providers, and/or family members and (2) described original research or reflection on research. We identified 23 manuscripts (see Figure 1).

### 3.2 Manuscript Characteristics

Manuscripts described studies with adults with developmental disabilities conducted in the United States (*n* = 8), the United Kingdom (*n* = 6), Ireland (*n* = 3), Australia (*n* = 2), Scotland (*n* = 2), and Finland (*n* = 1), with one study conducted both in Australia and the United States. A majority of manuscripts are retrospective reflections of research projects (*n* = 13). The remaining manuscripts used surveys (two manuscripts from the same dataset), focus groups (*n* = 4), interviews (*n* = 3), Delphi studies (one manuscript), and observations (*n* = 1). One manuscript described the development of a tool to support researchers responding to potential disclosures of abuse (see Table 1 for further information on manuscripts).

**TABLE 1:** Reviewed manuscripts.

Author(s) (year)	Country	Purpose	Manuscript type	Population and sample	Design/methods	Data source
Cithambaram et al. (2019)	Ireland	To describe inclusion of people with intellectual disability in end‐of‐life care research	Reflection	Adults with mild and moderate intellectual disability (*n* = 13)	Reflection on research project	Authors
Crook et al. (2015)	United Kingdom	To explore barriers to research in a community‐based disability services	Original research	Adults with intellectual disability (*n* = 5) and clinicians (*n* = 34)	Focus group with adults with intellectual disability and clinician surveys	Authors, people with intellectual disability, and clinicians
Drozd et al. (2021)	United Kingdom	To describe strategies for accessing, recruiting, and collecting qualitative data from adults with intellectual disability	Reflection	Adults with intellectual disability	Reflection on research project	Authors
Haines (2017)	United Kingdom	To explore ethical issues in qualitative research with people with intellectual disability	Reflection	People with profound intellectual disability (*n* = 5) and those supporting them	Qualitative study (observation, interviews, documents); critical ethnography	Authors
Hall (2013)	United States	To describe strategies used to include participants with intellectual disability in qualitative research	Reflection	Young adults with mild to moderate intellectual disability (*n* = 14)	Reflection on research project	Authors
Hills et al. (2020)	Australia/United States	To expand understanding on the use of interview for conducting research with autistic people	Reflection	Adults with level 3 autism (*n* = 7)	Reflection on research project	Authors and autistic adults
Johnson et al. (2011)	Australia	To explore the challenges and benefits in collecting participant observation data when researching interactions between adults with severe intellectual disability and their communication partners	Reflection	Adults (age 20–44) with severe intellectual disability (*n* = 6)	Reflection on research project	Authors
Kaley et al. (2019)	United Kingdom	To share methodological insights from a visual ethnographic study with people with intellectual disability	Reflection	People with intellectual disability (*n* = 7) and additional staff, family, paid carers, etc. (*n* = 13)	Reflection on research project	Authors
Kidney and McDonald (2014)	United States	To describe a qualitative study toolkit that respectfully includes people with intellectual/developmental disabilities in research	Reflection	Adults with intellectual and developmental disabilities	Reflection on research project	Authors
Manning (2010)	Australia	To share methodological approach used with people with intellectual disability	Reflection	Adults with intellectual disability (*n* = 80+)	Reflection on research project	Authors
Marshall and Tilley (2013)	Scotland	To record history on institutional and community‐based disability services	Reflection	People with intellectual disability (*n* = 35)	Reflection on research project	Authors
McDonald (2012)	United States	To explore how adults with intellectual and developmental disabilities want to be treated in research	Original research	Adults with intellectual and developmental disabilities (*n* = 16)	Interviews and focus groups	Adults with intellectual and developmental disabilities
McDonald et al. (2015)	United States	To describe community members' views on research with people with intellectual disability	Original research	Adults with intellectual disability (*n* = 24), family members and close friends of adults with intellectual disability (*n* = 12), and people who provide social services to adults with intellectual disability (*n* = 21)	Focus groups	Adults with intellectual disabilities, family members, and support providers
McDonald et al. (2016)	United States	To evaluate views on research safeguards	Original research	101 adults with intellectual disability 98 family/friends who provide nonpaid support 109 service providers 105 researchers 99 IRB members	Survey	Adults with intellectual disability, family and friends, service providers, disability researchers, and IRB members
McDonald et al. (2017)	United States	To study views on research harms for adults with intellectual disability	Original research	101 adults with intellectual disability 98 family/friends who provide nonpaid support 109 service providers 105 researchers 99 IRB members	Survey	Adults with intellectual disability, family and friends, service providers, disability researchers, and ethics review board members
Mietola et al. (2017)	Finland	To discuss ethical research practices for people with profound intellectual and multiple disabilities	Reflection	Adults with profound intellectual and multiple disabilities	Reflection on research project	Authors
Mulhall et al. (2020)	Northern Ireland	To reach consensus on methodological and practical challenges to conducting randomised controlled trials with adults with intellectual disability	Original research	Researchers (*n* = 22)	Delphi study	Disability researchers
Mulhall et al. (2021)	Northern Ireland	To explore challenges to conducting randomised controlled trials with adults with intellectual disability	Original research	International clinical trial experts (*n* = 12)	Semi‐structured interviews	Clinical trial experts
Northway et al. (2015)	United Kingdom	To explore involving people with intellectual disability in securing ethical approval for a participatory research project	Reflection	People with intellectual disability (*n* = 107)	Reflection on research project	Authors
Sammet Moring et al. (2019)	United States	To provide recommendations for responding to potential disclosures of abuse committed against participants with ID in a mixed‐methods pregnancy study	Original research	People with intellectual disability	Literature review, team consensus process, community partnership	Authors
Van der Weele and Bredewold (2021)	United Kingdom	To discuss shadowing as a method in intellectual disability research	Reflection	Study 1: Adults with intellectual disability (*n* = 28) and support workers (*n* = 13)	Reflection on research project	Authors
				Study 2: People with moderate to profound intellectual disability (*n* = 17)		
Wagner et al. (2020)	United States	To explore preferences for sharing electronic health records for research	Original research	Adults on the autism spectrum (*n* = 15), with fragile X syndrome (*n* = 13), or without neurodevelopmental disorder (*n* = 16)	Focus groups	Adults on the autism spectrum and with Fragile X
Watchman (2016)	Scotland	To identify methodological and ethical issues in a longitudinal study of the lived experiences of dementia in adults with Down syndrome	Original research	Adults with Down Syndrome (*n* = 3)	Interviews and participant observations	Authors

### 3.3 Data Extraction and Synthesis

The two first authors independently extracted data from Sections 2 and 3. We then systematically cross‐checked and discussed extracted data to verify relevance. When needed, we also reviewed information in introduction and discussion sections to determine if the extracted data were relevant. We then conducted thematic analysis (Braun and Clarke 2006) of content related to participant‐level risks and safeguards from methods and results sections. We used thematic analysis to identify commonalities among different types of data and to identify overarching commonalities across risks and safeguards, as discrete content analysis or a count of unique risks and safeguards may obfuscate underlying ideas driving perceived risks and enacted safeguards. After reviewing the data several times to inductively identify concepts (e.g., feeling upset, loss of confidentiality), we then categorised these concepts within commonly used risk categories (e.g., psychological risks, social risks) (National Commission for the Protection of Human Subjects of Biomedical and Behavioral Research 1979). We then coded each segment of data (i.e., main idea) by the inductively identified concepts and superordinate risk categories. Finally, we examined relationships among preliminary codes and their relationships to ideas in leading ethical principles (National Commission for the Protection of Human Subjects of Biomedical and Behavioral Research 1979). We used our deep exposure to sensitising concepts (i.e., ELSI) through our work on the broader project to examine commonalities across preliminary codes to identify themes. We returned to the data to verify themes and check for negative cases. Throughout, we refined codes and used procedures to strengthen rigour, including investigator triangulation (e.g., the third author reviewed all data extraction and coding) and an audit trail. Risks and safeguards were primarily described by the authors of reviewed manuscripts.

### 3.4 Positionality and Interpretive Rigour

K.M. has conducted research with adults with developmental disabilities and has been involved in research ethics as an area of empirical inquiry and practice for about two decades. She has experience designing and conducting community‐engaged social‐behavioural studies and in the review of the ethics of a range of research studies (including as an ethics board chair). MB has been a research assistant for three years, working on projects focused on ELSI in research with adults with developmental disabilities. AS has conducted research with adults with developmental disabilities for over 10 years. She has experience conducting and designing social‐behavioural studies, with a focus on peer‐delivered mental health interventions. We pursue research aligned with disability rights principles, including those emphasising inclusion and the right to dignity of risk. To enhance interpretive rigour, we: (1) developed a standardised review protocol; (2) conducted independent data extraction and analysis, systematically identifying discrepancies and resolving them via discussion; and (3) relied upon established bioethical principles.

## 4 Results

Our review identified several risks and safeguards (see Box 1 for an overview).

BOX 1 **Overview of Findings.**

1. Risks
  - Physical risks
    - Fatigue
    - Sensory sensitivities
  - Psychological risks
    - Distress and anxiety
    - Confusion
    - Burden
    - Frustration
  - Social risks
    - Relationships with other people
    - Societal perceptions
    - Relationships with researchers
  - Loss of privacy
  - Loss of confidentiality
2. Safeguards
  - Focus on rights, reflexive practice and community engagement
  - Reducing participant burden
    - Managing research duration
    - Provide advance information
    - Flexibility
  - Promoting privacy and confidentiality
    - Accessible consent/assent disclosures
    - Control over data elements
    - Provide choice
    - Confidentiality agreements for others present
    - Dissemination strategies that avoid identifiable data
    - Mandatory reporting procedures
  - Fostering psychological well‐being
    - Respect
    - Supports available
    - Enjoyable activities
  - Relational safeguards
    - Clear communication about relationships
    - Clarity regarding roles
    - Build rapport
    - Respect autonomy and competency
    - Learn about individualized forms of interaction/communication

### 4.1 Risks

Participant‐level risks are possible negative outcomes research participants may experience from participation. Manuscripts described risks associated with research participation of adults with developmental disabilities, including risks related to physical, relational, psychological, and social harms as well as a loss of privacy and confidentiality.

#### 4.1.1 Physical Risks

Manuscripts frequently described physical risks, such as fatigue, ability to tolerate procedures, and sensory sensitivity. Several manuscripts noted fatigue as related to the duration of research participation (Haines 2017; Hills et al. 2020; McDonald 2012; Mulhall et al. 2020, 2021; Van der Weele and Bredewold 2021). Participants with developmental disabilities noted that lengthy research activities may cause them to become “tired, confused, frustrated, develop headaches, or lose interest”, (McDonald 2012, 268) with another research team noting that participants expressed the need for sessions to conclude when they felt fatigued (Hills et al. 2020). A few researchers suggested that in addition to duration of procedures, the nature of procedures can contribute to fatigue, noting that not all participants may be able to tolerate every procedure on any given day, especially those involving equipment and devices that exacerbate sensory sensitivities (Mulhall et al. 2020).

#### 4.1.2 Psychological Risks

Authors emphasised the risk of psychological harms, which can manifest as distress or confusion due to the nature of a research topic, a change in location or routine, a lack of accommodation or support, or identification in dissemination products. Several manuscripts described potential distress when discussing sensitive topics in research such as death, violence, neglect, and abuse (Cithambaram et al. 2019; Manning 2010; Marshall and Tilley 2013; McDonald et al. 2015; Northway et al. 2015). However, two manuscripts suggested that what constitutes a sensitive topic can vary widely among individuals (McDonald et al. 2015; Northway et al. 2015), and some people with developmental disabilities reported that disclosing difficult, previously undisclosed emotions can also bring relief (McDonald et al. 2015). Importantly, sensitivities to research topics may emerge unexpectedly during data collection, making it difficult to fully anticipate topics that may be distressing (Northway et al. 2015).

Psychological risks related to feelings of burden, frustration, distress or anxiety could occur if research procedures resemble an exam or quiz (Van der Weele and Bredewold 2021), lead to changes in routines (Mulhall et al. 2021; Johnson et al. 2011), generate concerns about what participation would entail (McDonald et al. 2017), or require a long‐term commitment (Mulhall et al. 2021). Psychological risks may also occur if researchers do not provide emotional support and disability‐accommodations (McDonald 2012). Finally, one researcher expressed concerns that dissemination products may lead to psychological harm, as participants can “feel let down by their portrayal” (Haines 2017, 229) which could subsequently affect their self‐perception or how they are perceived by others.

#### 4.1.3 Social Risks

A few manuscripts described social risks that may occur, particularly regarding relationships. These relationships can be with researchers (discussed below) or the people around them. For example, one research team noted that their presence during observations led others to interact with participants less, resulting in reduced support and engagement in services during data collection (Johnson et al. 2011). In another manuscript, adults with developmental disabilities expressed concerns about a hypothetical scenario where a cure for autism discovered via research could be profit‐motivated and contribute to negative societal attitudes toward autistic people (Wagner et al. 2020).

Manuscripts noted potential negative psychological and social dynamics. For example, two manuscripts described situations when a researcher's presence as a novel person in someone's life created uncertainty, distress, and anxiety (Van der Weele and Bredewold 2021; Johnson et al. 2011). It is also possible that researchers' interactions with participants (e.g., tone of social‐behavioural voice) may cause distress (Johnson et al. 2011). Relatedly, adults with developmental disabilities have reported the importance of being treated with respect by researchers to avoid negative feelings, noting that being treated disrespectfully, as a label and not a person, or as a child, can lead to feelings of inferiority and self‐consciousness (McDonald et al. 2017, 2015; McDonald 2012).

A few manuscripts discussed relational risks concerning inaccurate or unmet relational expectations (Haines 2017; Van der Weele and Bredewold 2021; McDonald et al. 2015; Watchman 2016). For example, research methods involving prolonged interactions or that occur in participants' homes may foster relationship boundaries or understandings that are confusing with respect to the role of the researcher (e.g., questions of whether they are there to provide support) (Van der Weele and Bredewold 2021). These prolonged relationships can also create unmet expectations of continuation (Haines 2017; Van der Weele and Bredewold 2021; Watchman 2016), and/or feel like yet another transient relationship for those who already experience a high rate of them (McDonald et al. 2015; Watchman 2016).

#### 4.1.4 Loss of Privacy

Loss of privacy is highlighted as a significant concern in several manuscripts and is described as leading to discomfort and/or threats to dignity. Several manuscripts described how some research methods—such as shadowing, participant observation, in‐depth interviews, and video‐recording—can have the potential for significant invasion of privacy. This is especially true when data collection is done in the home, as it often involves entering personal spaces and observing intimate interactions (Haines 2017; Van der Weele and Bredewold 2021; Johnson et al. 2011; Watchman 2016; Hall 2013; Kaley et al. 2019; Mietola et al. 2017). Research that occurs in spaces that afford little privacy (e.g., when staff or family members frequently enter a participant's space and interrupt research activities) also can contribute to the loss of privacy (Hall 2013). Researchers noted that navigating privacy can be complex because some participants prefer not to have others present during data collection due to concerns about privacy, while others may want familiar people present to help them feel comfortable and better understand or participate (Hills et al. 2020; McDonald et al. 2015).

#### 4.1.5 Loss of Confidentiality

Loss of confidentiality can also create risks. Adults with developmental disabilities express concern that errors such as data breaches (Wagner et al. 2020), or dissemination practices such as the use of recordings or their personal information without permission (McDonald et al. 2017; Haines 2017; Marshall and Tilley 2013; Crook et al. 2015) may lead to a loss of confidentiality. One research team noted an unexpected potential breach when a participant wanted to co‐present findings, but doing so would reveal their identity (Drozd et al. 2021). Adults with developmental disabilities and researchers noted that loss of confidentiality may lead to backlash or repercussions from those who support them (e.g., the use of their personal information or negative information shared about services being used against them) (Manning 2010; McDonald et al. 2015; Wagner et al. 2020). Adults with developmental disabilities also noted feelings of betrayal and anger, and reluctance to participate in future studies when confidentiality is breached (Crook et al. 2015).

Doing research with adults with developmental disabilities can also create dilemmas regarding mandatory reporting of illegal activities such as abuse, neglect, or other illegal activities, which require researchers to disclose otherwise confidential information (McDonald et al. 2017; Haines 2017; Mulhall et al. 2020, 2021; Marshall and Tilley 2013; Sammet Moring et al. 2019). This may be more likely in research that gives researchers a high level of access to a participant's life (e.g., prolonged shadowing, interviewing at one's home) (Mulhall et al. 2020, 2021). While mandatory reporting requirements strive to prevent further harm, researchers noted that it can also generate new risks such as loss of relationships and re‐traumatisation from the response of the legal system, particularly when law enforcement responders lack the necessary knowledge and skills to interact with adults with developmental disabilities (Sammet Moring et al. 2019).

### 4.2 Safeguards

Safeguards are strategies that researchers can use to prevent risks or mitigate their impact on research participants. Manuscripts reviewed described a variety of safeguards including: a focus on rights as well as using reflexive practice and community engagement; reducing participant burden; securing privacy and confidentiality; and fostering psychological and relational well‐being.

#### 4.2.1 Focus on Rights, Reflexive Practice and Community Engagement

A few manuscripts described the importance of researchers prioritising the rights, well‐being, and safety of participants over data collection or research aims (Northway et al. 2015; Johnson et al. 2011; Mietola et al. 2017; Drozd et al. 2021), and general efforts to balance the aims of research and the ethical issues that might occur by giving primacy to ethical considerations (Johnson et al. 2011). Related, a few authors described the importance of continuous ethical reflection and sensitivity (Van der Weele and Bredewold 2021; Marshall and Tilley 2013; Kaley et al. 2019; Mietola et al. 2017), noting that researchers need to be aware of ways to minimise harm and be ready to adjust their methods as needed (Van der Weele and Bredewold 2021; Northway et al. 2015). Collaborations with self‐advocacy groups may yield effective guidance on how to resolve ethical issues (Marshall and Tilley 2013).

#### 4.2.2 Reducing Participant Burden

Several manuscripts suggested strategies to minimise participant burden. Strategies related to reducing fatigue included: reducing the length and frequency of research sessions (Mulhall et al. 2020, 2021), allowing breaks or stopping if participants become too tired (Watchman 2016). Additional strategies related to reducing burden were: collecting data across multiple sessions (Hills et al. 2020), providing questions in advance (Hills et al. 2020), and offering flexibility in the timing and location of data collection to avoid clashing with participants' other activities and to enhance comfort and willingness to participate (Hills et al. 2020; Hall 2013; Drozd et al. 2021).

#### 4.2.3 Promoting Privacy and Confidentiality

Several authors noted the importance of full, accessible consent disclosures and ensuring prospective participants understand privacy and confidentiality considerations, including how data will be used and limitations to confidentiality, at the time of enrollment (Haines 2017; Marshall and Tilley 2013; Northway et al. 2015; Crook et al. 2015; Drozd et al. 2021; Sammet Moring et al. 2019). Two authors described how they helped participants have greater control over their data and its use by giving participants choices during the consent process, including choosing pseudonyms and options for specific data collection elements and data use and withdrawal from the research (Manning 2010; Marshall and Tilley 2013). Adults with developmental disabilities agree that strategies to keep their information private are important (McDonald et al. 2016), with researchers adding that reminding participants of the right to stop participation during data collection is important (Cithambaram et al. 2019; McDonald et al. 2015).

To protect privacy, several authors described identifying boundaries and providing choice. For example, researchers identified boundaries for where or when observations will occur (e.g., in settings where others cannot overhear the participant) and will not occur (e.g., in places considered private such as bathrooms) occur (Van der Weele and Bredewold 2021; Johnson et al. 2011; Mietola et al. 2017). Two manuscripts noted that locations should be where researchers and participants can be visible to others but not overheard to maintain safety and comfort (Hall 2013; Drozd et al. 2021). Researchers also invited participants and/or their support persons to choose the locations of and content recorded during data collection (McDonald 2012; Van der Weele and Bredewold 2021; Hall 2013; Kaley et al. 2019; Mietola et al. 2017).

Several manuscripts also described safeguards to maintain participants' confidentiality. One manuscript suggested researchers should have those providing research participation support sign confidentiality forms (Hills et al. 2020). Five manuscripts discussed safeguards during dissemination, including de‐identifying data through the use of pseudonyms, not sharing direct quotes, removing or disguising identifiable information, and/or integrating various sources of data (Haines 2017; Van der Weele and Bredewold 2021; Marshall and Tilley 2013; Northway et al. 2015; Wagner et al. 2020). One manuscript noted the value of identifying in advance that some participants may elect to identify themselves in dissemination products (Drozd et al. 2021).

Two manuscripts discussed strategies for mandatory reporting. One manuscript discussed altering data collection approaches to avoid the need for mandatory reporting as they felt they could not ensure support would be available if needed, or alternatively having knowledgeable and experienced people available to handle disclosures (Northway et al. 2015). One manuscript outlined recommendations for conducting mandatory reporting, including responding to disclosures in ways that offer validation and support, avoid jumping to conclusions, and foster safety (including immediate actions for imminent danger). They also suggest interrupting the participant if disclosure starts to remind them of their obligation to report any allegations (Sammet Moring et al. 2019).

#### 4.2.4 Fostering Psychological Well‐Being

Ways to reduce psychological distress centered on approaches to data collection, making research enjoyable, and demonstrating respect. Researchers described how asking questions sensitively (e.g., in a generalised way) (Cithambaram et al. 2019) and only collecting information not previously shared or available (Watchman 2016) can help prevent psychological distress related to discussing distressing or private topics. One manuscript also discussed the benefits of having a familiar support person present during data collection to help participants feel more at ease (Haines 2017). However, two manuscripts also noted that preferences for the presence of a support person can vary, suggesting the need for researchers to individualise this strategy (McDonald et al. 2015, 2016). Another noted that integrating enjoyable activities into data collection can reduce anxiety (Kaley et al. 2019). Two manuscripts also described the importance of treating participants with respect. In one manuscript, participants with developmental disabilities expressed a desire for researchers to avoid being pushy, making mean or hurtful statements, or getting mad at them, with one participant sharing “Don't make fun of them ‘cause they're different, or, like, don't make fun of the way they speak (Claire)” McDonald (2012, 270). Similarly, another manuscript suggested researchers learn how to interact with adults with developmental disabilities and use respectful language when sharing research findings (McDonald et al. 2016).

When psychological distress occurs, several manuscripts noted ways to respond. A few manuscripts noted the importance of have support providers present or offering counselling services to help distressed participants (Cithambaram et al. 2019; Manning 2010; Northway et al. 2015; Johnson et al. 2011), and several others suggested researchers may also need to provide space for emotions, comfort or support (McDonald 2012; Cithambaram et al. 2019; Manning 2010; McDonald et al. 2015; Northway et al. 2015; Johnson et al. 2011; Kidney and McDonald 2014). Such support could also include taking a break or terminating research; researchers should plan ahead by informing participants of the options or creating a visual break board (McDonald 2012; Cithambaram et al. 2019; McDonald et al. 2015; Kidney and McDonald 2014). Lastly, one manuscript recommended researchers follow‐up with participants after data collection to ensure that no negative effects persisted (Cithambaram et al. 2019).

#### 4.2.5 Relational Safeguards

Many manuscripts described ways to address risks related to relationships with researchers. First, several manuscripts suggested providing clear, accessible, and concrete communication (e.g., using pictures and other visual indicators) about the nature and length of the researcher‐participant relationship (Haines 2017; McDonald et al. 2015, 2016; Johnson et al. 2011; Watchman 2016). Relatedly, two manuscripts indicated the importance of maintaining a clear researcher role (only breaking from this when observing malpractice or having knowledge germane to resolving a stressful situation) (Van der Weele and Bredewold 2021; Johnson et al. 2011). Another manuscript highlighted the value of planning a gradual withdrawal process to avoid causing participants distress but noted that real‐world constraints such as staff turnover can sometimes complicate doing so (Haines 2017).

Many authors, as well as people with developmental disabilities, also noted the importance of building rapport with participants, treating them with respect, and having skill in interacting and communicating with adults with developmental disabilities. They suggested using practices such as getting to know participants informally, making sure they are comfortable (perhaps achieved with individual planning), showing genuine interest in them, treating them with honesty (i.e., not making false promises, keeping their word) and as competent adults, respecting their autonomy, sharing their credentials, and using respectful language (Haines 2017; McDonald 2012; McDonald et al. 2015, 2016; Wagner et al. 2020; Hall 2013; Mietola et al. 2017; Kidney and McDonald 2014). One manuscript emphasised the importance of researchers learning, recognising, interpreting, and adapting to participants' interaction preferences to help build relationships (Mietola et al. 2017).

With regard to relationships with individuals other than researchers, researchers can adjust procedures to minimise interference with participants' social relationships by, for example, reducing notetaking during observations to avoid making interactions awkward (Johnson et al. 2011).

## 5 Discussion

We conducted a qualitative systematic review of risks and safeguards in social‐behavioural research with adults with developmental disabilities. Concerns for increased vulnerability to risks are one rationale put forward to exclude adults with developmental disabilities from research (Spong and Bianchi 2018; McDonald et al. 2022, 2009), and safeguards can promote or restrict autonomy. As such, this work holds critical implications for fostering trust in science among adults with developmental disabilities and research policy and practice, and the availability of scientific discoveries to promote social and health equity.

We identified risks associated with research participation of adults with developmental disabilities and safeguards recommended or used for research with them (see Box 1). By and large, these are the same risks—and at what appears to be more or less the same rate of occurrence, or at least not substantially a heightened rate—and safeguards as are commonly described and recommended for research with any participant population, underscoring the similarities among people with and without disabilities. Critically, these findings provide further evidence that adults with developmental disabilities may not experience different types or rates of harm in social‐behavioural research. These observations further suggest that rather than having elevated psychological vulnerability, people with developmental disabilities have noteworthy psychological resilience (McDonald et al. 2009).

Our findings also reveal some considerations that may be unique to the research participation of adults with developmental disabilities. For example, researchers may need to specifically attend to burden, as the need for the tolerability of research procedures, breaks, and multiple sessions may be especially important. Researchers may also consider their relationships with participants, given that people with developmental disabilities have many fluctuating personal and professional relationships in their lives due to service structures. These approaches may include finding concrete ways to demonstrate the specific nature of their role, maintaining boundaries tailored to the nature of the relationship, and planning for the end of relationships. Of note, researchers might consider whether it is possible or desirable to maintain any kind of longer‐term relationship, and how their interactions convey a presumption of competence and respect toward participants. Similarly, researchers may need to plan carefully for securing privacy and confidentiality, which may be more easily threatened by data collection methods or settings (e.g., a reliance on qualitative methods in private settings) and service and housing systems' routine dismissal of individuals' rights to privacy and confidentiality (McDonald et al. 2024) Related, researchers may wish to deploy additional strategies and potentially assurances that consent information is fully understood. Ethics review boards will similarly need to learn about these areas so their reviews can integrate these considerations.

Safeguards identified in this review point to the continued importance of choice and autonomy in the context of research. Many approaches to limit burden (psychological risk), breaches of privacy and confidentiality, and social risks centre on giving participants control of their data and the data collection process. Researchers should identify possibilities a priori to ensure that they can offer a set of options that optimises choices without compromising rigour. Further, because many adults with disabilities have limited opportunities for autonomy and choice in their lives, researchers may find that they need to develop rapport and an environment of psychological safety as baseline conditions for participants to exert control over their participation experience and data. Finally, within the constraints of a study design, participants should have the opportunity to revise their initial decisions (e.g., change how data is used, location of data collection, timing of breaks, support provided, etc.). Ethics review boards can help foster choice and autonomy by reviewing applications through this lens.

### 5.1 Implications

As noted by others (Iltis et al. 2013; Labott and Johnson 2004), risk identification is complex and controversial. Risks play an important role in informing inclusion, with respect to eligibility, as well as conditions for research participation. We use our findings and other relevant work—including the perspectives of people with developmental disabilities—to inform recommendations for how to balance real and perceived vulnerability with responsible inclusion.

Because the evidence base remains relatively small and linked to a somewhat restricted range of methods, we encourage researchers to systematically collect quantitative information on observed risks and the acceptability and effectiveness of safeguards from the perspectives of adults with developmental disabilities. These findings can be shared widely, including with ethics review boards, people with developmental disabilities, and gatekeepers who influence research participation. Clarity on actual risks and effective safeguards can inform research policy and practice. Increased availability of reliable information about risks and safeguards should decrease negative impacts of ableism that can lead to widespread exclusion from research or use of safeguards that restrict rights (McDonald et al. 2022). Specifically, this enhanced evidence base will help researchers proactively identify possible concerns, systematically assess for risks specific to their research protocol, address anticipated concerns in protocols, and communicate plans for risk mitigation to ethics review boards and prospective research participants (Iltis et al. 2013).

Our findings suggest it may be prudent that researchers assume information covered under mandatory reporting requirements can emerge. Therefore, researchers should plan for ways to prevent its occurrence, ensure participants understand limitations to confidentiality, and approach mandatory reporting with respect for the person and efforts to decrease negative impacts on them (Sammet Moring et al. 2019; Oschwald et al. 2014). People with developmental disabilities should have the opportunity to refine these recommended practices, and ethics review board members should learn about these recommendations so their reviews can reflect them.

When developing safeguards, we urge researchers to examine whether they resonate with disability rights principles that emphasise the right to inclusion and control over decisions affecting one's life and presume competence. Again, part of translating these ideas into practice will require that people with developmental disabilities have the opportunity to inform best practices, and ethics review board members learn about their appropriateness. As noted, community‐engaged research is an especially effective way to inform risk identification and assessment and create responsive safeguards that are perceived as effective and respectful from the perspective of adults with developmental disabilities. Although less focused on herein, finding ways to enhance informed, voluntary, and ongoing consent provides individuals the information they need and the opportunity to make their own decisions—a key idea essential to realising the ethical principles emphasising the rights of people to make their own decisions.

Lastly, we suggest researchers depart from a position of primacy of ethical and moral commitments to individuals' rights and well‐being over scientific pursuits. Relatedly, commitment to continued reflexive practice may be an essential strategy to enhance the responsible inclusion of adults with developmental disabilities in research. Discussions with an array of partners and interested parties may shed new insight into understanding possible negative consequences of research participation and acceptable, effective ways to prevent their occurrence and lessen their impact.

### 5.2 Limitations

Our review has important limitations to consider, including a relatively small number of articles reviewed, heavy reliance on researchers' reflections, and the exclusion of grey literature. Because we reviewed published research, each study had undergone review and approval by the required ethics committees. We do not necessarily know what changes were required to yield these approvals and how researchers felt about the final research protocol. Also of note, ethics review board approval requires an analysis of risks and benefits; our review did not address this analysis, as manuscripts rarely included information on benefits, therefore leaving us without new insight into risks and benefits analysis. Future research may include a review of IRB applications, consent materials, or information collected from researchers to study the role of benefits and risk/benefit ratios. We also note that our emphasis on participant‐level risks leaves out group and community risks (e.g., interpretations of or acting on findings in ways that cause group harm), which are equally important, yet receive less attention overall (Oschwald et al. 2014). We urge researchers and ethics review boards to also attend to the prevention and mitigation of group and community risks; community‐engaged research is one way to lessen these, though the well‐being of community research partners also merits attention. We underscore the importance of the views of adults with developmental disabilities themselves, yet seldom did reviewed manuscripts include information about these views. Lastly, we remind readers of our exclusive focus on social –behavioural research; other research approaches may generate different risks and safeguards critical to considering.

## 6 Conclusions

The responsible inclusion of adults with developmental disabilities is necessary to yield scientific discoveries that can advance social and health equity. Realising this outcome requires responsible risk identification and implementation of acceptable, effective safeguards. These critical aspects of scientific integrity have far‐reaching negative implications, from fostering feelings of disrespect to restricting rights for individuals to increasing harm to groups; outcomes that threaten trust, decrease volunteerism, and dampen the promise of scientific advances. We encourage researchers to strive to do more than just prevent risks and reduce their negative impacts: Instead, our goal should be to make a commitment to fostering positive experiences for research participants, leaving them having felt valued and respected, and having enjoyed their experience and opportunity to contribute to scientific discoveries.

## Ethics Statement

The authors have nothing to report.

## Conflicts of Interest

The authors declare no conflicts of interest.

## Supporting information


**Supplemental Table 1.** Database Search Strings.

## Data Availability

The data that support the findings of this study are available from the corresponding author upon reasonable request.
