# Supplementary material for: Risks and Safeguards in Social‐Behavioural Research With Adults With Developmental Disabilities: A Qualitative Systematic Review
Source: J Appl Res Intellect Disabil. 2025 Mar 7;38(2):e70031. doi: 10.1111/jar.70031 (PMC11886930; doi:10.1111/jar.70031)
Supplement: Supplementary file 1 — Supplemental Table 1. Database Search Strings. [file JAR-38-e70031-s001.docx]

**Supplemental Table 1: Database Search Strings**

| **Search string** | **Filters used** | **Date of search** | | **# of results** | |
| --- | --- | --- | --- | --- | --- |
|  |  | **Original search** | **String + gatekeeping** | **Original search** | **String + gatekeeping** |
| CINAHL | | | | | |
| "developmental disab*" OR "intellectual disab*” OR “Schinzel-Giedion Syndrome” OR “Cockayne Syndrome” OR “Coffin-Siris Syndrome” OR “Cri-Du-Chat Syndrome” OR “De Barsy Syndrome” OR “De Lange Syndrome” OR “Down Syndrome” OR “Gaucher Disease” OR “Kernicterus” OR “Kinky Hair Syndrome” OR “Kleefstra Syndrome” OR “Phenylketonuria” OR “Prader-Willi Syndrome” OR “Rett Syndrome” OR “Rubinstein-Taybi Syndrome” OR “WAGR Syndrome” OR “Williams Syndrome” OR “Adrenoleukodystrophy” OR “Coffin-Lowry Syndrome” OR “Fragile X Syndrome” OR “Mucopolysaccharidosis II" OR “autistic disorder*” OR “Asperger* Syndrome” OR “Pervasive Developmental Disorder-Not Otherwise Specified” OR “learning disorder*” OR “Mental retardation, X-linked”  AND  “Ethic*” OR “ethical issue*” OR “social issue*” OR “Measurement Issues and Assessments” OR “Social Values“ OR “legal issue*” OR “Decision Making, Ethical” OR “Privacy” OR “Confidential*” OR “Duty to Warn” OR “Genetic privacy” OR “competence {Legal}” OR “Guardianship, Legal” OR “Special Populations” OR “Abuse reporting” OR “Mandat* reporting” OR “Bioethic*”  AND  “Community-based practice research” OR “Community based practice research” OR “Community engaged research” OR “Community-engaged research” OR “action research” OR “inclusive research” OR “patient-engaged research” OR “patient engaged research” OR “patient centered outcomes research” OR “patient-centered outcomes research” OR “Patient participation” OR  “Research ethic*” OR “confidentiality {Research}” OR “Conflict of Interest {Research}” OR “Protection of Human Subjects” OR “Consent {Research}” OR “Institutional Review” OR “Researcher-Subject Relations” OR "Consent" OR "Assent" | English; peer-reviewed; 1/1/2009-search date | June 2, 2022 | March 31, 2023 | 269 | 316 |
| ERIC | | | | | |
| “Developmental disab*” OR “Intellectual Disab*” OR “Down Syndrome” OR “Mild Intellectual Disability” OR Moderate Intellectual Disability” OR “Severe Intellectual Disability” OR “Autism” OR “Autis*” OR “Pervasive Developmental Disorder” OR “Asperger Syndrome” OR “Fragile X” OR “Fragile X Syndrome” OR “Rett Syndrome” OR “Mental Retardation” OR "Fetal Alcohol Syndrome” OR “Cerebral Palsy”  AND  “Ethical issue*” OR “Social issue*” OR “Legal issue*” OR Justice OR “Ethic*” OR “Conflict* of Interest” OR “Informed Consent” OR “Privacy” OR “Confidential*”  AND  “Community-based practice research” OR “Community based practice research” OR “Community engaged research” OR “Community-engaged research” OR “participatory action research” OR “inclusive research” OR “patient-engaged research” OR “patient engaged research” OR “patient centered outcomes research” OR “patient-centered outcomes research” OR “Client Participation” OR “Research Ethic*” OR “Research subject relations” OR "Consent*" OR "Assent*" | English; Peer-reviewed; 1/1/2009-date of search | June 2, 2022 | March 31, 2023 | 53 | 59 |
| PSYCInfo | | | | | |
| "Developmental disab*" OR "Intellectual development disorder*" OR "Anencephaly" OR "Crying Cat syndrome" OR "Tay Sachs Disease" OR "Down's syndrome" OR "Autism spectrum disorder*" OR "autistic trait*" OR "Learning disorder*" OR "Learning disab*" OR "Fragile X Syndrome" OR "Rett syndrome"  AND  "professional ethic*" OR "boundary crossing" OR "boundary violation*" OR "conflict* of interest" OR "duty to protect" OR "duty to warn" OR "dual relationship*" OR "professional standards" OR "ethic*" OR "Social issue*" OR "social disadvantage*" OR "social equality" OR "social integration" OR "unemployment" OR "child welfare" OR "human rights" OR "justice" OR "Legal process*" OR "guardian*" OR "informed consent" OR "abuse reporting" OR "mandat* reporting"  AND  "Community-based practice research" OR "Community based practice research" OR "Community engaged research" OR "Community-engaged research" OR "action research" OR "inclusive research" OR "patient-engaged research" OR "patient engaged research" OR "patient centered outcomes research" OR "patient-centered outcomes research" OR "Patient participation" OR "experimental ethic*" OR "research subject relations" OR "consent*" OR "assent*" | Peer-reviewed; 1/1/2009-date of search | June 2, 2022 | March 31, 2023 | 235 | 255 |
| PubMed | | | | | |
| (("Cri du Chat Syndrome" OR "De Lange Syndrome" OR "Down Syndrome" OR "Adrenoleukodystrophy" OR "Coffin Lowry Syndrome" OR "Fragile X Syndrome" OR "Mucopolysaccharidosis II" OR "Pyruvate Dehydrogenase Complex Deficiency Disease" OR "Rett Syndrome" OR "Prader Willi Syndrome" OR "Rubinstein Taybi Syndrome" OR "Trisomy 13 Syndrome" OR "WAGR Syndrome" OR "Williams Syndrome" OR "Pervasive Child Development Disorders" OR "autism spectrum disorder" OR "Asperger Syndrome" OR "Glycogen Storage Disease Type IIB" OR "Lesch Nyhan Syndrome" OR "Menkes Kinky Hair Syndrome" OR "Developmental disab*" OR "Intellectual Disab*" OR "Mental* Retard*" OR "Autistic Disorder*" OR "Learning Disab*") AND ("Institutional ethics" OR "Professional ethics" OR "Personal Autonomy" OR "Relational Autonomy" OR "Social Justice" OR "Scientific Misconduct" OR "Conflict of Interest" OR "Bioethic*" OR "Bioethical issue*" OR "Ethical Review*" OR "Ethic* Consultation*" OR "Ethics Committee*" OR "Research Ethics Committee*" OR "Ethical issue*" OR "Legal issue*" OR "Social issue*" OR "Principle Based Ethic*" OR "Beneficen*" OR "Ethic*")) AND ("Community-Based Participatory Research" OR "Community Based Participatory Research" OR "Citizen Science" OR "Community engaged research" OR "Community-engaged research" OR "action research" OR "inclusive research" OR "patient-engaged research" OR "patient engaged research" OR "patient centered outcomes research" OR "patient-centered outcomes research" OR "patient participation" OR "Research Ethic*" OR "Research censorship" OR "Helsinki Declaration" OR "Therapeutic Equipoise" OR "Therapeutic Misconception" OR "Scientific misconduct" OR "Consent*" OR "Assent") AND ((2009:2022[pdat]) AND (english[Filter])) | English; Peer-reviewed; 1/1/2009-date of search | June 2, 2022 | March 31, 2023 | 1127 | 1176 |
| ProQuest | | | | | |
| ((ab("Community-Based Practice Research" OR "Community Based Practice Research" OR "Patient research participation" OR "Participatory action research" OR "inclusive research" OR "patient-engaged research" OR "patient engaged research" OR "patient centered outcomes research" OR "patient-centered outcomes research" OR "research ethic*" OR "research subject relation*" OR “consent*” OR “assent*”) OR MAINSUBJECT.EXACT("Human subjects")) AND (ab(("fragile x syndrome" OR fxs OR "rett syndrome" OR "down syndrome") OR "Developmental disab*" OR "Cerebral Palsy" OR "learning disab*" OR "Intellectual disab*" OR "Fetal alcohol syndrome" OR Autis* OR "Learning difficult*" OR "Fragile X syndrome" OR "Rett syndrome" OR "Down Syndrome" OR "Intellectual disab*") OR (MAINSUBJECT.EXACT("Cerebral palsy") OR MAINSUBJECT.EXACT("Autism") OR (MAINSUBJECT.EXACT("Intellectual disabilities") OR MAINSUBJECT.EXACT("Developmental disabilities")) OR MAINSUBJECT.EXACT("Fetal alcohol syndrome"))) AND (ab("Ethical issue*" OR "Social Issue*" OR "Equality" OR "Human Rights" OR "Injustice" OR "Legal Issue" OR "Legal Ethic*" OR "Research ethic*" OR "Research subject relations" OR "Ethic*" OR "Bioethic*" OR "Conflict* of interest" OR "Professional ethic*" OR "Professional misconduct" OR "Duty to warn" OR "Professional relationship*") OR (MAINSUBJECT.EXACT("Legal ethics") OR (MAINSUBJECT.EXACT("Ethics") OR MAINSUBJECT.EXACT("Bioethics") OR MAINSUBJECT.EXACT("Professional ethics") OR MAINSUBJECT.EXACT("Conflicts of interest")) OR MAINSUBJECT.EXACT("Professional misconduct") OR MAINSUBJECT.EXACT("Duty to warn") OR MAINSUBJECT.EXACT("Professional relationships")))) AND (stype.exact("Scholarly Journals") AND pd(20090101-20220606)) | English; Peer-reviewed; 1/1/2009-date of search | June 8^th^, 2022 | March 31, 2023 | 284 | 315 |
| ASSIA | | | | | |
| ((ab("Community-Based Practice Research" OR "Community Based Practice Research" OR "Patient research participation" OR "Participatory action research" OR "inclusive research" OR "patient-engaged research" OR "patient engaged research" OR "patient centered outcomes research" OR "patient-centered outcomes research" OR "research ethic*" OR "research subject relation*" OR “consent*” OR “assent*”) OR MAINSUBJECT.EXACT("Human subjects")) AND (ab(("fragile x syndrome" OR fxs OR "rett syndrome" OR "down syndrome") OR "Developmental disab*" OR "Cerebral Palsy" OR "learning disab*" OR "Intellectual disab*" OR "Fetal alcohol syndrome" OR Autis* OR "Learning difficult*" OR "Fragile X syndrome" OR "Rett syndrome" OR "Down Syndrome" OR "Intellectual disab*") OR (MAINSUBJECT.EXACT("Cerebral palsy") OR MAINSUBJECT.EXACT("Autism") OR (MAINSUBJECT.EXACT("Intellectual disabilities") OR MAINSUBJECT.EXACT("Developmental disabilities")) OR MAINSUBJECT.EXACT("Fetal alcohol syndrome"))) AND (ab("Ethical issue*" OR "Social Issue*" OR "Equality" OR "Human Rights" OR "Injustice" OR "Legal Issue" OR "Legal Ethic*" OR "Research ethic*" OR "Research subject relations" OR "Ethic*" OR "Bioethic*" OR "Conflict* of interest" OR "Professional ethic*" OR "Professional misconduct" OR "Duty to warn" OR "Professional relationship*") OR (MAINSUBJECT.EXACT("Legal ethics") OR (MAINSUBJECT.EXACT("Ethics") OR MAINSUBJECT.EXACT("Bioethics") OR MAINSUBJECT.EXACT("Professional ethics") OR MAINSUBJECT.EXACT("Conflicts of interest")) OR MAINSUBJECT.EXACT("Professional misconduct") OR MAINSUBJECT.EXACT("Duty to warn") OR MAINSUBJECT.EXACT("Professional relationships")))) AND (stype.exact("Scholarly Journals") AND pd(20090101-20220606)) | English; Peer-reviewed; 1/1/2009-date of search | June 8^th^, 2022 | March 31, 2023 | 31 | 32 |
| Scopus | | | | | |
| {developmental disab*} OR {developmental* impair*} OR {developmental* handicap*} OR {developmental* delay*} OR {intellectual disab*} OR {autis*} OR {learning disab*} OR {learning difficulties} OR {mental* retard*} OR {down syndrome} OR {fragile X syndrome} OR {rett syndrome} AND {ethical issue*} OR {social issue*} OR {legal issue*} OR {ethics} AND {community based participatory research} OR {community engaged research} OR {participatory action research} OR {inclusive research} OR {patient engaged research} OR {patient centered outcomes research} OR {patient participation} OR {research ethics} OR {Research subject relations} OR {consent*} OR {assent*} AND { LIMIT-TO { LANGUAGE , "English" } | English; Peer-reviewed; 1/1/2009-date of search | June 8, 2022 | March 31, 2023 | 763 | 840 |
| Web of Science | | | | | |
| “developmental disab*” OR “developmental* impair*” OR “developmental* handicap*” OR “developmental* delay*” OR “Intellectual disab*” OR “autis*” OR “autism spectrum disorder” OR “learning disab*” OR “learning difficulties” OR “mental* retard*” OR “Down syndrome” OR “Fragile X syndrome” OR “Rett syndrome”  AND  “ethical issue*” OR “social issue*” OR “legal issue*” OR “ethics”  AND  “Community-Based Participatory Research" OR “Community based participatory research” OR “community engaged research” OR “Community-engaged research” OR “Participatory action research” OR “inclusive research” OR “Patient-engaged research” OR “Patient-centered outcomes research” OR “Patient centered outcomes research” OR “Patient participation” OR “research ethics” OR “Research subject relations” OR “consent*” OR “assent*” | English; Peer-reviewed; 1/1/2009-6/2/2022 | June 8, 2022 | March 31, 2023 | 340 | 479 |

**Table Y: Original Database Search Strings + Gatekeeping Term**

| **Database** | **Search string** | **Filters used** | **Date of search** | **# of results** | **# of articles added** |
| --- | --- | --- | --- | --- | --- |
| CINAHL | "developmental disab*" OR "intellectual disab*” OR “Schinzel-Giedion Syndrome” OR “Cockayne Syndrome” OR “Coffin-Siris Syndrome” OR “Cri-Du-Chat Syndrome” OR “De Barsy Syndrome” OR “De Lange Syndrome” OR “Down Syndrome” OR “Gaucher Disease” OR “Kernicterus” OR “Kinky Hair Syndrome” OR “Kleefstra Syndrome” OR “Phenylketonuria” OR “Prader-Willi Syndrome” OR “Rett Syndrome” OR “Rubinstein-Taybi Syndrome” OR “WAGR Syndrome” OR “Williams Syndrome” OR “Adrenoleukodystrophy” OR “Coffin-Lowry Syndrome” OR “Fragile X Syndrome” OR “Mucopolysaccharidosis II" OR “autistic disorder*” OR “Asperger* Syndrome” OR “Pervasive Developmental Disorder-Not Otherwise Specified” OR “learning disorder*” OR “Mental retardation, X-linked”  AND  “Ethic*” OR “ethical issue*” OR “social issue*” OR “Measurement Issues and Assessments” OR “Social Values“ OR “legal issue*” OR “Decision Making, Ethical” OR “Privacy” OR “Confidential*” OR “Duty to Warn” OR “Genetic privacy” OR “competence {Legal}” OR “Guardianship, Legal” OR “Special Populations” OR “Abuse reporting” OR “Mandat* reporting” OR “Bioethic*”  AND  “Community-based practice research” OR “Community based practice research” OR “Community engaged research” OR “Community-engaged research” OR “action research” OR “inclusive research” OR “patient-engaged research” OR “patient engaged research” OR “patient centered outcomes research” OR “patient-centered outcomes research” OR “Patient participation” OR  “Research ethic*” OR “confidentiality {Research}” OR “Conflict of Interest {Research}” OR “Protection of Human Subjects” OR “Consent {Research}” OR “Institutional Review” OR “Researcher-Subject Relations” OR "Consent" OR "Assent" OR “Gatekeep*” | English; peer-reviewed; 1/1/2009-3/31/2023 | 3/31/2023 | 316 | 37 |
| ERIC | “Developmental disab*” OR “Intellectual Disab*” OR “Down Syndrome” OR “Mild Intellectual Disability” OR Moderate Intellectual Disability” OR “Severe Intellectual Disability” OR “Autism” OR “Autis*” OR “Pervasive Developmental Disorder” OR “Asperger Syndrome” OR “Fragile X” OR “Fragile X Syndrome” OR “Rett Syndrome” OR “Mental Retardation” OR "Fetal Alcohol Syndrome” OR “Cerebral Palsy”  AND  “Ethical issue*” OR “Social issue*” OR “Legal issue*” OR Justice OR “Ethic*” OR “Conflict* of Interest” OR “Informed Consent” OR “Privacy” OR “Confidential*”  AND  “Community-based practice research” OR “Community based practice research” OR “Community engaged research” OR “Community-engaged research” OR “participatory action research” OR “inclusive research” OR “patient-engaged research” OR “patient engaged research” OR “patient centered outcomes research” OR “patient-centered outcomes research” OR “Client Participation” OR “Research Ethic*” OR “Research subject relations” OR "Consent*" OR "Assent*" OR “Gatekeep*” | English; Peer-reviewed; 1/1/2009-3/31/2023 | 3/31/2023 | 59 | 5 |
| PSYCInfo | "Developmental disab*" OR "Intellectual development disorder*" OR "Anencephaly" OR "Crying Cat syndrome" OR "Tay Sachs Disease" OR "Down's syndrome" OR "Autism spectrum disorder*" OR "autistic trait*" OR "Learning disorder*" OR "Learning disab*" OR "Fragile X Syndrome" OR "Rett syndrome"  AND  "professional ethic*" OR "boundary crossing" OR "boundary violation*" OR "conflict* of interest" OR "duty to protect" OR "duty to warn" OR "dual relationship*" OR "professional standards" OR "ethic*" OR "Social issue*" OR "social disadvantage*" OR "social equality" OR "social integration" OR "unemployment" OR "child welfare" OR "human rights" OR "justice" OR "Legal process*" OR "guardian*" OR "informed consent" OR "abuse reporting" OR "mandat* reporting"  AND  "Community-based practice research" OR "Community based practice research" OR "Community engaged research" OR "Community-engaged research" OR "action research" OR "inclusive research" OR "patient-engaged research" OR "patient engaged research" OR "patient centered outcomes research" OR "patient-centered outcomes research" OR "Patient participation" OR "experimental ethic*" OR "research subject relations" OR "consent*" OR "assent*" OR “Gatekeep*” | Peer-reviewed; 1/1/2009-3/31/2023 | 3/31/2023 | 255 | 13 |
| PubMed | (("Cri du Chat Syndrome" OR "De Lange Syndrome" OR "Down Syndrome" OR "Adrenoleukodystrophy" OR "Coffin Lowry Syndrome" OR "Fragile X Syndrome" OR "Mucopolysaccharidosis II" OR "Pyruvate Dehydrogenase Complex Deficiency Disease" OR "Rett Syndrome" OR "Prader Willi Syndrome" OR "Rubinstein Taybi Syndrome" OR "Trisomy 13 Syndrome" OR "WAGR Syndrome" OR "Williams Syndrome" OR "Pervasive Child Development Disorders" OR "autism spectrum disorder" OR "Asperger Syndrome" OR "Glycogen Storage Disease Type IIB" OR "Lesch Nyhan Syndrome" OR "Menkes Kinky Hair Syndrome" OR "Developmental disab*" OR "Intellectual Disab*" OR "Mental* Retard*" OR "Autistic Disorder*" OR "Learning Disab*") AND ("Institutional ethics" OR "Professional ethics" OR "Personal Autonomy" OR "Relational Autonomy" OR "Social Justice" OR "Scientific Misconduct" OR "Conflict of Interest" OR "Bioethic*" OR "Bioethical issue*" OR "Ethical Review*" OR "Ethic* Consultation*" OR "Ethics Committee*" OR "Research Ethics Committee*" OR "Ethical issue*" OR "Legal issue*" OR "Social issue*" OR "Principle Based Ethic*" OR "Beneficen*" OR "Ethic*")) AND ("Community-Based Participatory Research" OR "Community Based Participatory Research" OR "Citizen Science" OR "Community engaged research" OR "Community-engaged research" OR "action research" OR "inclusive research" OR "patient-engaged research" OR "patient engaged research" OR "patient centered outcomes research" OR "patient-centered outcomes research" OR "patient participation" OR "Research Ethic*" OR "Research censorship" OR "Helsinki Declaration" OR "Therapeutic Equipoise" OR "Therapeutic Misconception" OR "Scientific misconduct" OR "Consent*" OR "Assent" OR “Gatekeep*”) AND ((2009:2022[pdat]) AND (english[Filter])) | English; Peer-reviewed; 1/1/2009-3/31/2023 | 3/31/2023 | 1176 | 44 |
| ProQuest | ((ab("Community-Based Practice Research" OR "Community Based Practice Research" OR "Patient research participation" OR "Participatory action research" OR "inclusive research" OR "patient-engaged research" OR "patient engaged research" OR "patient centered outcomes research" OR "patient-centered outcomes research" OR "research ethic*" OR "research subject relation*" OR “consent*” OR “assent*” OR “gatekeep*”) OR MAINSUBJECT.EXACT("Human subjects")) AND (ab(("fragile x syndrome" OR fxs OR "rett syndrome" OR "down syndrome") OR "Developmental disab*" OR "Cerebral Palsy" OR "learning disab*" OR "Intellectual disab*" OR "Fetal alcohol syndrome" OR Autis* OR "Learning difficult*" OR "Fragile X syndrome" OR "Rett syndrome" OR "Down Syndrome" OR "Intellectual disab*") OR (MAINSUBJECT.EXACT("Cerebral palsy") OR MAINSUBJECT.EXACT("Autism") OR (MAINSUBJECT.EXACT("Intellectual disabilities") OR MAINSUBJECT.EXACT("Developmental disabilities")) OR MAINSUBJECT.EXACT("Fetal alcohol syndrome"))) AND (ab("Ethical issue*" OR "Social Issue*" OR "Equality" OR "Human Rights" OR "Injustice" OR "Legal Issue" OR "Legal Ethic*" OR "Research ethic*" OR "Research subject relations" OR "Ethic*" OR "Bioethic*" OR "Conflict* of interest" OR "Professional ethic*" OR "Professional misconduct" OR "Duty to warn" OR "Professional relationship*") OR (MAINSUBJECT.EXACT("Legal ethics") OR (MAINSUBJECT.EXACT("Ethics") OR MAINSUBJECT.EXACT("Bioethics") OR MAINSUBJECT.EXACT("Professional ethics") OR MAINSUBJECT.EXACT("Conflicts of interest")) OR MAINSUBJECT.EXACT("Professional misconduct") OR MAINSUBJECT.EXACT("Duty to warn") OR MAINSUBJECT.EXACT("Professional relationships")))) AND (stype.exact("Scholarly Journals") AND pd(20090101-20220606)) | English; Peer-reviewed; 1/1/2009-3/31/2023 | 3/31/2023 | 315 | 27 |
| ASSIA | ((ab("Community-Based Practice Research" OR "Community Based Practice Research" OR "Patient research participation" OR "Participatory action research" OR "inclusive research" OR "patient-engaged research" OR "patient engaged research" OR "patient centered outcomes research" OR "patient-centered outcomes research" OR "research ethic*" OR "research subject relation*" OR “consent*” OR “assent*” OR “Gatekeep*”) OR MAINSUBJECT.EXACT("Human subjects")) AND (ab(("fragile x syndrome" OR fxs OR "rett syndrome" OR "down syndrome") OR "Developmental disab*" OR "Cerebral Palsy" OR "learning disab*" OR "Intellectual disab*" OR "Fetal alcohol syndrome" OR Autis* OR "Learning difficult*" OR "Fragile X syndrome" OR "Rett syndrome" OR "Down Syndrome" OR "Intellectual disab*") OR (MAINSUBJECT.EXACT("Cerebral palsy") OR MAINSUBJECT.EXACT("Autism") OR (MAINSUBJECT.EXACT("Intellectual disabilities") OR MAINSUBJECT.EXACT("Developmental disabilities")) OR MAINSUBJECT.EXACT("Fetal alcohol syndrome"))) AND (ab("Ethical issue*" OR "Social Issue*" OR "Equality" OR "Human Rights" OR "Injustice" OR "Legal Issue" OR "Legal Ethic*" OR "Research ethic*" OR "Research subject relations" OR "Ethic*" OR "Bioethic*" OR "Conflict* of interest" OR "Professional ethic*" OR "Professional misconduct" OR "Duty to warn" OR "Professional relationship*") OR (MAINSUBJECT.EXACT("Legal ethics") OR (MAINSUBJECT.EXACT("Ethics") OR MAINSUBJECT.EXACT("Bioethics") OR MAINSUBJECT.EXACT("Professional ethics") OR MAINSUBJECT.EXACT("Conflicts of interest")) OR MAINSUBJECT.EXACT("Professional misconduct") OR MAINSUBJECT.EXACT("Duty to warn") OR MAINSUBJECT.EXACT("Professional relationships")))) AND (stype.exact("Scholarly Journals") AND pd(20090101-20220606)) | English; Peer-reviewed; 1/1/2009-3/31/2023 | 3/31/2023 | 32 | 0 |
| Scopus | {developmental disab*} OR {developmental* impair*} OR {developmental* handicap*} OR {developmental* delay*} OR {intellectual disab*} OR {autis*} OR {learning disab*} OR {learning difficulties} OR {mental* retard*} OR {down syndrome} OR {fragile X syndrome} OR {rett syndrome} AND {ethical issue*} OR {social issue*} OR {legal issue*} OR {ethics} AND {community based participatory research} OR {community engaged research} OR {participatory action research} OR {inclusive research} OR {patient engaged research} OR {patient centered outcomes research} OR {patient participation} OR {research ethics} OR {Research subject relations} OR {consent*} OR {assent*} OR {gatekeep*} AND { LIMIT-TO { LANGUAGE , "English" } | English; Peer-reviewed; 1/1/2009-3/31/2023 | 3/31/2023 | 840 | 84 |
| Web of Science | “developmental disab*” OR “developmental* impair*” OR “developmental* handicap*” OR “developmental* delay*” OR “Intellectual disab*” OR “autis*” OR “autism spectrum disorder” OR “learning disab*” OR “learning difficulties” OR “mental* retard*” OR “Down syndrome” OR “Fragile X syndrome” OR “Rett syndrome”  AND  “ethical issue*” OR “social issue*” OR “legal issue*” OR “ethics”  AND  “Community-Based Participatory Research" OR “Community based participatory research” OR “community engaged research” OR “Community-engaged research” OR “Participatory action research” OR “inclusive research” OR “Patient-engaged research” OR “Patient-centered outcomes research” OR “Patient centered outcomes research” OR “Patient participation” OR “research ethics” OR “Research subject relations” OR “consent*” OR “assent*” OR “gatekeep*” | English; Peer-reviewed; 1/1/2009-3/31/2023 | 3/31/2023 | 479 | 27 |
